# Acculturation and School Adjustment of Immigrant Youth in Six European Countries: Findings from the Programme for International Student Assessment (PISA)

**DOI:** 10.3389/fpsyg.2017.00649

**Published:** 2017-05-04

**Authors:** Maja K. Schachner, Jia He, Boris Heizmann, Fons J. R. Van de Vijver

**Affiliations:** ^1^Inclusive Education, University of PotsdamPotsdam, Germany; ^2^Department of Methodology and Statistics, Tilburg UniversityTilburg, Netherlands; ^3^Department Data Archive for the Social Sciences, GESIS – Leibniz Institute for the Social SciencesCologne, Germany; ^4^Department of Culture Studies, Tilburg UniversityTilburg, Netherlands; ^5^Faculty of Economics and Management Sciences, North-West UniversityPotchefstroom, South Africa; ^6^School of Psychology, University of Queensland, BrisbaneQLD, Australia

**Keywords:** adolescent immigrants, acculturation, multicultural policy, cross-cultural comparison, school adjustment

## Abstract

School adjustment determines long-term adjustment in society. Yet, immigrant youth do better in some countries than in others. Drawing on acculturation research (Berry, 1997; Ward, 2001) and self-determination theory (Ryan and Deci, 2000), we investigated indirect effects of adolescent immigrants’ acculturation orientations on school adjustment (school-related attitudes, truancy, and mathematics achievement) through school belonging. Analyses were based on data from the Programme for International Student Assessment from six European countries, which were combined into three clusters based on their migrant integration and multicultural policies: Those with the most supportive policies (Belgium and Finland), those with moderately supportive policies (Italy and Portugal), and those with the most unsupportive policies (Denmark and Slovenia). In a multigroup path model, we confirmed most associations. As expected, mainstream orientation predicted higher belonging and better outcomes in all clusters, whereas the added value of students’ ethnic orientation was only observed in some clusters. Results are discussed in terms of differences in acculturative climate and policies between countries of settlement.

## Introduction

School adjustment, including academic achievement, is among the most important acculturation outcomes of immigrant youth and an important developmental outcome in adolescence (Sam et al., 2006; Motti-Stefanidi et al., 2012). Yet, there are large differences in adjustment outcomes between countries of settlement. Whereas in some studies, mainly in North America, youth from some immigrant groups outperformed youth representing the cultural majority in a so-called “immigrant paradox” (García Coll et al., 2012), a recent meta-analysis in Europe confirmed that adolescents of immigrant background are more at risk of lower psychological and academic adjustment compared to their native peers (Dimitrova et al., 2016). A gap in academic achievement, with immigrant students lagging behind their native peers, has also been identified in large-scale educational surveys, such as the Programme for International Student Assessment (PISA; OECD, 2006, 2012). The existence and magnitude of this gap across countries seems to be associated with differences in multicultural climate and policies (Arikan et al., in press).

An increasing body of comparative research has been conducted to explain between-country differences in the school adjustment of immigrant youth (Martin et al., 2012; Teltemann and Schunck, 2016). Yet, most studies focused on macro-level conditions (such as differences in educational systems) and achievement-related outcomes, whereas acculturative processes and psychological aspects of school adjustment are usually neglected. In this study, we investigated adolescent immigrants’ acculturation orientations and school adjustment (school-related attitudes, truancy and mathematics achievement) across different countries of settlement in Europe using the 2012 PISA data. Integrating acculturation research (Berry, 1997; Ward, 2001; Motti-Stefanidi et al., 2012) and self-determination theory (Ryan and Deci, 2000), we develop and argue for a conceptual model where acculturation orientations are associated with better school adjustment indirectly through strengthening students’ sense of school belonging.

### Acculturation and Adjustment of Immigrant Youth

Acculturation refers to dealing with psychological stress, acquiring new skills, and developing a sense of identity and belonging during cultural transition or when navigating between different cultural groups (Ward, 2001). Acculturation orientations refer to the orientation toward ethnic (heritage) culture and mainstream (host) culture, including the respective identity components. They form the attitudinal component of the acculturation process and facilitate psychological (“feeling well”) and sociocultural (“doing well”) adjustment. Integration (i.e., an orientation toward both ethnic and mainstream culture) has long been perceived as the most adaptive strategy (Berry, 1997). However, depending on the context and the particular outcome (psychological or sociocultural), a stronger orientation toward the ethnic or mainstream culture can be more adaptive (Motti-Stefanidi et al., 2012; Ward, 2013).

As for the link between acculturation orientations and outcomes, research from Germany revealed that students’ mainstream orientation was associated with better sociocultural outcomes (e.g., achievement, mainstream language skills, and mainstream friends) and better psychological outcomes (e.g., higher academic self-concept, less disruptive behavior and delinquency at school), while students’ ethnic orientation was only associated with better psychological outcomes (Schachner et al., 2016, in press). Yet, a recent review suggests that the relation between immigrant students’ acculturation orientations and school adjustment may differ between countries and may be moderated by between-country variation in the acculturative climate (Makarova and Birman, 2015). At the same time, students’ sense of school belonging may act as a mediator between acculturation orientations and adjustment.

### Belonging as a Mediator between Acculturation Orientations and Adjustment

The need to belong has long been recognized as one of the most important psychological needs in humans (Maslow, 1943; Bowlby, 1977; Ainsworth, 1989; Baumeister and Leary, 1995). In self-determination theory (Ryan and Deci, 2000), which has been frequently applied in educational settings (Niemiec and Ryan, 2009), relatedness and psychological belonging are linked to motivation and personal growth. Research with immigrants and members of ethnic minorities shows that identification with and orientation toward their ethnic culture can promote a sense of social belonging (Ward, 2001), notably in the face of discrimination and exclusion by members of the mainstream society (Branscombe et al., 1999). Meanwhile, the orientation toward and identification with the mainstream society may facilitate a sense of belonging in a predominantly mainstream context like school (Güngör, 2007), especially if there is a high expectation by members of the mainstream society that immigrants and members of ethnic minorities should assimilate to the mainstream culture.

School belonging has been defined as “the extent to which students feel personally accepted, respected, included, and supported by others in the school social environment” (Goodenow, 1993, p. 80). It is also sometimes treated as the emotional component of school engagement, which is linked to other, more behavioral forms of engagement as well as achievement (Fredricks et al., 2004). Research has consistently shown that immigrant students experience a lower sense of belonging at school than their mainstream peers. They also experience a steeper decline in belonging through adolescence (Li and Lerner, 2011). This phenomenon, sometimes referred to as the belonging gap, partly explains the achievement gap between immigrant (or ethnic minority) and non-immigrant (or ethnic majority) students (Motti-Stefanidi and Masten, 2013; Voight et al., 2015).

A high level of school belonging has been identified as a major protective factor associated with lower levels of delinquency in adolescent immigrants (Titzmann et al., 2008). School belonging was also found to mediate the relationship between perceived school context (e.g., classroom climate, student-teacher relations) and academic outcomes, such as achievement (Wang and Holcombe, 2010), intrinsic motivation (Byrd, 2015), and psychological school adjustment (Schachner et al., unpublished) of culturally diverse students (for a review, see Eccles and Roeser, 2011). A study with Latino students in the United States confirmed that students who felt more connected with their teachers and their school were also more motivated to attend school, which was in turn associated with better achievement (Close and Solberg, 2008). A recent study from Belgium further suggests that a sense of relatedness with the school and the teacher had a stronger effect on achievement for Turkish immigrant students than for their national mainstream peers (Coskan et al., 2015). It is therefore of great importance to identify individual and policy-level conditions which are associated with higher school belonging and engagement, specifically amongst immigrant students.

### Country-Level Conditions for Acculturation and School Adjustment

Climate and policies in the country of settlement form an important macro-context for the acculturation and adjustment of immigrants more generally (Ward, 2013; Ward and Geeraert, 2016), and adolescent immigrants in particular (Motti-Stefanidi et al., 2012). Indeed, there are systematic variations in immigrants’ acculturation orientations and ethnic identity between countries of settlement, which have been linked to multicultural climate and policies in these countries (Yaǧmur and Van de Vijver, 2012). The association between immigrant students’ orientations toward ethnic and mainstream culture and acculturative outcomes in the school context also seems to differ between countries. A recent review suggests that a stronger mainstream and weaker ethnic orientation (i.e., assimilation) can be more beneficial for school adjustment in assimilation-oriented countries (Makarova and Birman, 2015). A study based on data from PISA 2009 confirmed that in Germany, which could be taken to exemplify such a country, assimilation was associated with better reading competences compared to integration (Edele et al., 2013).

In this study we compare acculturation orientations and their effects on school belonging and adjustment across six European countries, namely Belgium, Denmark, Finland, Italy, Portugal, and Slovenia. These were the only European countries where acculturation orientations were measured and there was sufficient immigrant youth data available. Serbia was also initially included, but had to be excluded due to the extremely low reliability of some of the measures in this sample. The countries chosen also differ in terms of their immigrant populations as well as their multicultural climate and policies.

The policy differences we discuss here refer to differences in the Migrant Integration Policy Index (MIPEX) and the Multiculturalism Policy Index (MCP). The policies that are captured here not only determine the institutional stance toward immigrants in terms of immigrant rights, they also have a bearing on how immigration is viewed by the non-immigrant population (Heizmann, 2016). Both indicator sets also tap into education and schools as institutions. The reliability and validity of both indices was established via a cross-validation with other integration-related policy measures such as access to citizenship (Helbling, 2013; Koopmans, 2013).

The MIPEX measures policy support for access of immigrants in institutional settings and equal rights by way of an expert survey (Huddleston et al., 2011). Specifically, the index taps into areas like family reunion, labor market mobility, access to long-term residence and citizenship, as well as education. In the domain of education, the index also captures aspects of the school system that make education more or less accessible for immigrant students, such as whether there are support programs geared toward the specific needs of immigrant students and their parents or whether teachers are required to be trained on dealing with cultural diversity in the classroom. Experts rate the presence of such programs by the extent to which the respective policy is implemented on three levels, for instance whether training is required, offered extensively or offered only in a very minimal fashion in teacher education.

The MCP on the other hand measures active recognition of diversity and cultural differences in the form of special group rights as well as a general endorsement of multiculturalism (Banting and Kymlicka, 2013) based on an original analysis of policy documents and government communications. Specific dimensions include constitutional, legislative or parliamentary affirmation of multiculturalism, funding of ethnic group organizations to support cultural activities, funding of bilingual education or mother-tongue instruction, as well as the adoption of multiculturalism in the school curriculum, for example via the representation of diversity in school books. For each component of this index there is also a three-level rating indicating full, partial or no adoption of the respective policy aspect.

There are clear differences between the countries in the policies vis-à-vis diversity as well as their immigrant populations (see **Table 1**). Belgium, Finland, Portugal, and to some extent also Italy have relatively favorable institutional conditions, promoting access and equal rights for immigrants, whereas Denmark and Slovenia have more restrictive policies (Huddleston et al., 2011). A similar pattern arises for multicultural policies. While Belgium and Finland strongly endorse such policies, there is only moderate support in Portugal and Italy. Denmark does not follow this approach at all, rendering Denmark the least supportive environment in comparison to the other countries. There is no information available for Slovenia regarding political multiculturalism (Banting and Kymlicka, 2013), but the very low MIPEX scores strongly suggest this to be an unsupportive surrounding as well. Belgium and Finland are the most supportive countries across both policy indicators, and Italy and Portugal can be regarded as moderately supportive due to their lower commitment to multiculturalism.

**Table 1:** Countries, policies, and immigration.

	MIPEX 2010 (0 to 100)		MCP 2010		Immigration	
Country	Summary score	Education score	Summary score (0 to 8)	School curriculum score (0 to 1)	Five Largest Immigrant Groups in 2010 (sorted by size in descending order)	Overall Share of Immigrants (%)
Belgium	67	66	5.5	0.5	Italy, France, Netherlands, Morocco, Spain	10.2
Denmark	53	51	0	0	Germany, Turkey, Poland, Iraq, Sweden	9.2
Finland	69	63	6	1	Russian Federation, Sweden, Estonia, Somalia, Iraq	4.6
Italy	60	41	1.5	0.5	Romania, Albania, Morocco, Germany, Switzerland	7.9
Portugal	79	63	3.5	0.5	Angola, Brazil, France, Mozambique, Cape Verde	8.1
Slovenia	49	24	N/A	N/A	Bosnia and Herzegovina, Croatia, Serbia, Former Yugoslav Republic of Macedonia, Germany	11.1

*Sources: Migrant Integration Policy Index: http://www.mipex.eu, Multiculturalism Policy Index: www.queensu.ca/mcp/, immigrant statistics: United Nations Population Division (United Nations database, POP/DB/MIG/Stock/Rev.2013). Higher values on the policy indices indicate more supportive environments. The Belgian immigrant group ranking is based on citizenship; all other rankings are based on country of birth*.

The composition of the immigrant populations in each country in terms of countries of origin to some extent mirrors these policy differences: In Belgium, Finland, Italy, and Portugal there are several large groups from culturally more distant countries (i.e., countries that differ more from the receiving country in terms of cultural values and level of development; Ward and Geeraert, 2016). However, Denmark has traditionally followed a restrictive stance toward immigration in terms of policies, even though there is a notable presence of immigrants with a Turkish or Iraqi background. Furthermore, for Portugal, a large share of immigrants originates from Portuguese-speaking countries, most of them former colonies. In Slovenia most of the immigrant population are from neighboring countries of the former Yugoslav Republic. Although these countries comprise different ethnic groups, between-country differences in terms of cultural values and overall level of economic and political development are small.

We expect that the extent to which ethnic and mainstream orientations are associated with higher school belonging and adjustment varies between countries. A mainstream orientation is generally thought to be beneficial in all countries and independent of immigrant-group specific characteristics, since schools are institutions firmly embedded in the respective mainstream culture. Nonetheless, we expect that having a mainstream orientation is especially important in societies where societal institutions and school systems (as reflected in the MIPEX) are less adapted to immigrant needs, and multicultural climate and policies (as reflected in the MCP) are not supportive of cultural diversity and heritage culture maintenance. In contrast, an ethnic orientation would be more beneficial where heritage culture maintenance is supported by attendant multicultural policies. The additional positive effect of ethnic orientation on school adjustment has also been found as a consequence of positive multicultural policies in schools (Schachner et al., 2016).

### The Present Study

Most research on psychological and specifically acculturative processes underlying immigrant students’ psychological and academic adjustment at school has focused on proximal, micro-level contexts like the family or the school (e.g., Schachner et al., 2014b, 2016). Some studies have focussed on more distal, macro-level conditions and their link with achievement-related outcomes amongst immigrant students but have not looked into underlying processes, such as acculturative processes (Martin et al., 2012). In the present study we attempt to bridge this gap and enhance our understanding of how societal contexts interact with individual-level acculturative processes, shaping psychological and academic outcomes in immigrant students. Our findings may thereby ultimately contribute to evidence-based policy recommendations.

Using a large data set from the 2012 PISA and including immigrant students from six European countries, we study the relationship between acculturation orientations, school belonging (i.e., feeling socially integrated at school), and a range of school adjustment outcomes, namely school-related attitudes (i.e., perceived usefulness of school), truancy and mathematics achievement. We focused on these outcomes as they reflect both academic (achievement) and psychological (school-related attitudes, truancy) aspects of school adjustment. We chose the domain of mathematics for our achievement measure as this was the main focus in PISA 2012 and also as we expected it to be less culture and language dependent than reading. Further, as psychological school adjustment may be manifested differentially in attitudinal and behavioral domains for boys and girls (Schachner et al., in press), we provide a more holistic picture of psychological adjustment by including both an attitudinal (school-related attitudes) and a behavioral (truancy) measure.

We focus on two research questions, namely (1) how the between-country differences in the association between acculturation conditions and school belonging reflect differences in the acculturative climate and policies in the countries of settlement; and (2) if the effects of acculturation orientations on school adjustment outcomes of immigrant students are mediated by students’ sense of school belonging across countries of settlement. Specifically, we test the following hypotheses:

H1: In countries high on multicultural policies, both mainstream and ethnic orientation of immigrant students are positively associated with sense of school belonging, whereas in countries low on multicultural policies, only the mainstream orientation is positively associated with sense of school belonging. The association between mainstream orientation and school belonging is expected to be stronger in countries lower on multicultural policies.
H2: Across countries of settlement, a higher sense of belonging is associated with more favorable school-related attitudes, less truancy, and higher mathematics achievement, thereby mediating the effects of acculturation orientations on school adjustment.

Generational status, family educational background, and the use of the test language at home have emerged as significant individual-level predictors of mathematics achievement of immigrant students in research drawing on previous cycles of PISA (e.g., Martin et al., 2012). Therefore, these variables were included as covariates. Research with first-generation immigrants in the US has further confirmed that social relations and the sense of belonging at school largely mediate the effect of these background variables on achievement-related outcomes (Suárez-Orozco et al., 2009). We expected that students representing the first generation of immigrants, from less educated families and who do not speak the test language at home to experience less belonging and to be less adjusted at school. We also included students’ perceptions of cultural distance as a covariate to better accommodate for differences in the immigrant populations in the six countries. Groups which are perceived to be more different from the mainstream society also tend to have a lower social status (Hagendoorn, 1995). A study with early adolescent immigrants in Germany shows that their perceptions of cultural distance are significantly related to country-level variables such as values or the level of development (Schachner et al., 2014a). A greater cultural distance often implies more perceived discrimination through the mainstream society and has been associated with a higher ethnic and lower mainstream orientation as well as lower levels of (school) adjustment (Galchenko and Van de Vijver, 2007; Schachner et al., in press). We expect that students with a higher perceived cultural distance experience less belonging and be less adjusted at school.

## Materials and Methods

The 2012 PISA student survey measured performance of 15-year-olds in reading, mathematics, and science (with the focus in 2012 being on mathematics) in different cultural contexts. All students took a background questionnaire and a subset of the cognitive test of different combinations that lasted 2 h. APA ethical standards have been followed. The student questionnaire, data, manual, procedure and the assessment frameworks are available on the OECD website in the PISA 2012 technical report (OECD, 2014).

### Sample

A total of 5,334 students with an immigrant background in the six European countries are included in the present study. Immigrant background involves country of birth of the students and/or at least one of their parents outside the country of assessment. The demographics of these students are presented in **Table 2**. The share of males was about half in all countries and ranged from 47 to 54%. The percentage of students stating that they used the test language (i.e., language of the mainstream culture) at home varied from 18% in Finland to 64% in Portugal. The generational status of students in the sample also differs from country to country. First-generation immigrants refer to those students born outside the country of assessment and whose parents were also born in another country. All other students in our sample are second-generation immigrants who were born in the country of assessment but with at least one parent born in another country. In Belgium and Finland, the percentage of first- and second-generation immigrant students was about the same; in Denmark and Slovenia, there were more second-generation immigrant students than first-generation, whereas the reverse was true for Italy and Portugal. Unfortunately, information on students’ country of origin was recorded very differently across countries. Measures ranged from broad categories like “country of assessment” vs. “other country” in some countries to much more fine-grained categories of the most frequent immigrant groups in the country of assessment in other countries. Due to this inconsistency, it was not possible to include this information in our analyses. Our covariates (educational background, generational status, test language used at home, and perceived cultural distance) were therefore the only way of getting at differences between immigrants in the six countries.

**Table 2:** Descriptive statistics of sample characteristics, control variables, and main study variables.

	Country					
Descriptive statistics	Belgium	Denmark	Finland	Italy	Portugal	Slovenia
Sample characteristics and control variables						
*N*	871	1393	1077	1229	340	424
Male (%)	51%	54%	52%	53%	52%	47%
Educational background [*M* (*SD*)]	2.61 (1.46)	2.37 (1.27)	2.52 (1.32)	2.39 (1.25)	2.49 (1.30)	2.04 (1.18)
Perceived cultural distance [*M* (*SD*)]	6.71 (2.39)	7.86 (2.13)	6.31 (2.26)	6.60 (2.21)	6.11 (2.13)	6.27 (2.09)
Test language at home (%)	39%	42%	18%	29%	64%	36%
First-generation immigrants (%)	51%	29%	51%	78%	60%	30%
Main study variables						
Mainstream orientation [*M* (*SD*)]	12.07 (2.85)	11.75 (2.99)	13.23 (2.46)	13.33 (2.27)	12.74 (2.56)	12.13 (2.51)
Ethnic orientation [*M* (*SD*)]	12.73 (2.58)	12.72 (2.44)	12.92 (2.64)	12.47 (2.46)	12.23 (2.65)	13.28 (2.41)
School belonging [*M* (*SD*)]	24.86 (4.08)	25.84 (4.01)	25.43 (4.05)	23.87 (3.77)	25.10 (3.82)	25.47 (3.95)
Mathematics achievement^a^ [*M* (*SD*)]	459.41 (92.35)	440.13 (78.04)	444.72 (85.68)	460.19 (90.86)	456.59 (94.73)	441.83 (86.83)
Positive attitude toward School [*M* (*SD*)]	12.03 (2.21)	12.14 (2.10)	12.69 (2.12)	12.23 (2.11)	12.38 (2.06)	12.35 (2.25)
Truancy [*M* (*SD*)]	1.25 (.41)	1.36 (.49)	1.47 (.52)	1.46 (.50)	1.59 (.62)	1.44 (.56)
Difference acculturation orientations^b^						
*F* (df1, df2)	28.68^∗∗^ (1, 835)	69.67^∗∗^ (1, 1312)	2.64 (1, 1043)	67.09^∗∗^ (1, 1194)	19.47^∗∗^ (1, 330)	62.73^∗∗^ (1, 408)
Partial eta squared	0.03	0.05	0.00	0.05	0.06	0.13

*Country means are based on sum scores of individual respondents without missing values in respective variables. ^a^Means reported are the average mean of the five plausible values. ^b^Within-subject contrast by country. *^∗∗^ p* < 0.01*.

### Measures

All measures were part of the PISA 2012 student survey. Information beyond what is provided below can be retrieved from the technical report (OECD, 2014). Sum scores were calculated for scales comprising multiple items.

*Educational Background* of the student was measured with the number of books at home as a proxy for family educational background. Our sample comprised immigrant students from many countries of origin in six different host countries. We therefore opted against a global index of socio-economic status and chose a measure that was considered most valid and comparable across countries, and least dependent on the education system of a particular country. Response options ranged from 1 (*0–10 books*) to 6 (*more than 500 books*).

*Perceived Cultural Distance* between countries of origin and the host country was measured with three items, tapping into the perceived similarity of cultural values, mother and teacher behavior. Responses ranged from 1 (*Strongly Agree*) to 4 (*Strongly Disagree*). The reliability ranged from 0.67 to 0.78, with a mean of 0.74. Perceived cultural distance was largest in Finland, followed by Belgium and Denmark, and lowest in Slovenia.

*Acculturation Orientations* were measured with eight items: four items for the *Mainstream (host culture) Orientation* (e.g., participate in mainstream culture celebrations), and the other four items for the *Ethnic (heritage culture) Orientation* (e.g., spend time with ethnic friends). The response options ranged from 1 (*Strongly Agree*) to 4 (*Strongly Disagree*). The reliability of *Mainstream Orientation* across the six countries ranged from 0.71 to 0.88, with a mean of 0.84, and that of *Ethnic Orientation* ranged from 0.79 to 0.85, with a mean of 0.82. Both scale scores were reverse-coded, so that a higher score on either scale indicated a stronger orientation.

*School Belonging* reflected the extent to which students felt they belonged to and were socially integrated at school. It was measured with nine items with the same 4-point Likert scale as acculturation orientations. Sample items included “Other students seem to like me” and “I feel awkward and out of place in my school” (reversed). According to the Technical Report (OECD, 2014), Item 8 “Things are ideal at school” was understood very differently across countries, thus this item was deleted from the scale. The reliability of the 8-item scale across countries ranged from 0.79 to 0.84, with an average of 0.81.

*Positive Attitude toward School – Learning Outcomes* captured to what extent students experienced school as being useful for their current and future lives. It was measured with a 4-item scale with the same response options. Sample items included “school has taught me things which could be useful in a job” and “school has been a waste of time” (reversed). The reliability across countries ranged from 0.67 to 0.73, with an average of 0.69.

*Truancy* was measured with three self-report items on the frequency of skipping a whole school day, skipping classes within a school day, and being late for school within the last 2 weeks from 1 (*None*) to 4 (*Five or more times*). The reliability ranged from 0.59 to 0.74, with a mean of 0.63.

*Mathematics Achievement* was measured with different subsets of the cognitive test (rotation design). With systematic variations of the cognitive items across student groups, item difficulty could be determined based on item response theory (IRT) and the data were then scaled using an IRT model (which allows for missing data due to the incomplete design), from which student math achievement scores were derived as plausible values. Plausible values are imputed values that resemble individual test scores and have approximately the same distribution as the latent trait being measured. Five plausible values of math achievement for each student were produced, thus standard analyses with math achievement should be performed on each of the plausible values and the results of each analysis should be combined (OECD, 2009; Rutkowski et al., 2010).

## Results

We report the findings in two parts: We first describe the measurement invariance of target scales, which is the basis for the hypotheses tests, and the descriptive statistics of the study variables. We then report the multigroup path model where we tested our hypotheses. For both the measurement models and the path model, the model fit was mainly evaluated by fit indexes including CFI (above 0.90, and change from the less constrained to the more constrained smaller than 0.01), and RMSEA (below 0.06). Chi-square values were also reported, but given the large sample size, these values were less ideal to judge model fit.

The measurement invariance of the scales, including the acculturation orientations, school belonging, and positive attitude toward school were checked across the six countries in AMOS (Arbuckle, 2010). For all the scales, metric invariance was supported (**Table 3**), which was sufficient for the comparison of the relationship between these variables across countries. Scalar invariance, which is required if we were to compare means across countries, was not supported for any of the scales. Due to these invariance issues, significance tests of differences between ethnic and mainstream orientation were only carried out within countries. Country means and standard deviations of all scales as well as within-subject contrasts for acculturation orientations are displayed in **Table 2**. In all countries except Finland, the mean scores of the two acculturation orientations differed significantly. Ethnic and mainstream orientations were most different with effect sizes indicating medium to large effects in Italy and Portugal (stronger mainstream orientation), Denmark and Slovenia (both stronger ethnic orientation).

**Table 3:** Measurement invariance of the scales.

Scale	Model	Chi-square	*df*	CFI	RMSEA
Cultural distance	Unconstrained	Saturated model			
	*Metric*	*31.07*	*12*	*1.00*	*0.01*
	Scalar	634.03	30	0.91	0.04
Host orientation	Unconstrained	659.26	14	0.93	0.09
	*Metric*	*745.31*	*32*	*0.93*	*0.06*
	Scalar	1332.28	56	0.87	0.06
Heritage orientation	Unconstrained	552.59	14	0.93	0.08
	*Metric*	*594.84*	*32*	*0.93*	*0.06*
	Scalar	1021.34	56	0.88	0.06
School belonging	Unconstrained	102.87	119	0.91	0.04
	*Metric*	*1102.32*	*161*	*0.90*	*0.03*
	Scalar	1722.49	209	0.84	0.04
Attitude toward school	Unconstrained	38.93	14	0.98	0.02
	*Metric*	*71.26*	*32*	*0.97*	*0.02*
	Scalar	364.87	56	0.80	0.03

*Most restrictive model with acceptable fit is printed in italics*.

In the next step we were interested in the associations between our study variables (see Appendix 1, Supplementary Data Sheet for correlations across the whole sample). We tested the path model with acculturation orientations predicting all three outcomes through a stronger sense of school belonging employing multigroup structural equation modeling in AMOS. Family educational background, generational status, test language used at home as well as perceived cultural distance were included as control variables. We initially specified effects of all covariates on school belonging. Yet, in order to improve model fit and as the link between family educational background and achievement is well-established, we allowed for a direct effect of family educational background (the indirect effect was non-significant and was therefore excluded from the final model). Given the missing values in the data, we used full information maximum likelihood estimation. The unconstrained model revealed a good fit, χ^2^(108) = 433.69, *p* < 0.001, CFI = 0.91, RMSEA = 0.02, and there were no further above-threshold modification indices, suggesting that the relations we had specified in our model were adequate. The unconstrained model also confirmed that there was considerable variation between countries in the effects of acculturation orientations on school belonging.

In order to make the model more parsimonious we looked for patterns in these associations in the unconstrained model. Our assumption was that associations between acculturation orientations and school belonging would differ as a function of migrant integration and multicultural policies, as discussed in section “Country-Level Conditions for Acculturation and School Adjustment.” Supporting this assumption, a similar pattern of associations emerged in the two countries with the most supportive policies (Belgium and Finland), the two countries with moderately supportive policies (Italy and Portugal), and the two countries with the most unsupportive policies (Denmark and Slovenia).

**FIGURE 1:**
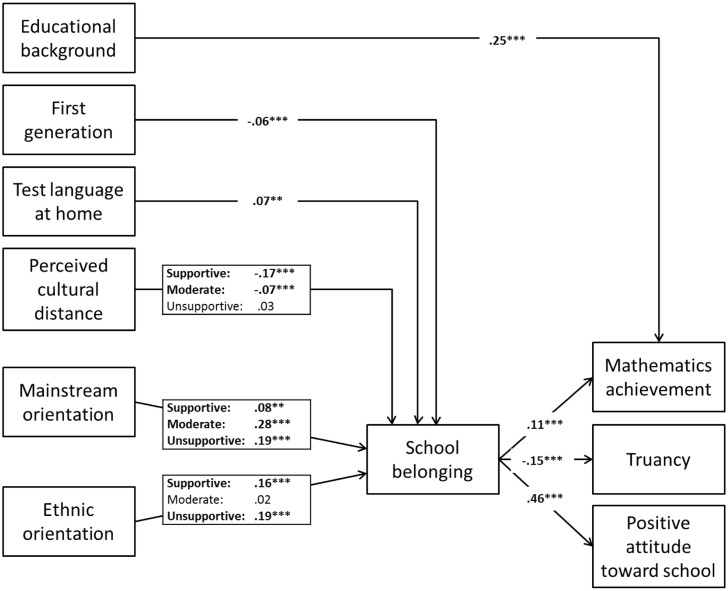
**Multigroup partial structural weights model with standardized coefficients**. Effects where only one coefficient is reported are constrained to be equal across all countries; the others were constrained by cluster. To accommodate slightly different coefficients due to sample size fluctuations, average coefficients across countries were reported. Supportive = countries with highly supportive policies (Belgium, Finland); Moderate = countries with moderately supportive policies (Italy, Portugal); Unsupportive = countries with unsupportive policies (Denmark, Slovenia). Significant paths are printed in bold. ^∗∗^*p* < 0.01 and ^∗∗∗^*p* < 0.001.

We therefore clustered countries accordingly (highly supportive, moderately supportive, and unsupportive policy context) and then constrained effects of the acculturation orientations to be the same for the two countries within a cluster. All other paths were constrained to be equal across all countries. In order to improve model fit, we also allowed for differential effects of perceived cultural distance between clusters. The resulting partial structural weights model revealed a good fit, χ^2^(147) = 523.03, *p* < 0.001, CFI = 0.90, RMSEA = 0.02, and a change in CFI smaller than 0.01. This suggests that constraining paths to be equal within clusters did not significantly decrease model fit and our clustering solution therefore was adequate. We tested the final model five times with all five plausible values of mathematics achievement. Very similar model fit and regression weights were achieved for each of the plausible values, thus only one model is reported. The final model as well as coefficients for individual paths is displayed in **Figure 1**.

In line with previous research, a higher educational background was associated with better mathematics achievement. Being a first-generation immigrant was associated with a slightly lower sense of school belonging, whereas speaking the test language at home was associated with a slightly higher sense of school belonging. Students who perceived more cultural distance experienced less school belonging in countries with highly supportive policies. Surprisingly, this effect was smaller in countries with moderately supportive policies and could not be observed at all in countries with unsupportive policies.

As expected, a stronger mainstream orientation was associated with a stronger sense of school belonging in all countries, but this effect was stronger in countries with moderate or unsupportive policies. An additional positive effect of ethnic orientation could only be observed in the highly supportive policy cluster, but – unexpectedly – also in the unsupportive policy cluster. Finally, school belonging was a significant predictor of all three school adjustment outcomes in the expected direction, with the strongest effect on positive attitudes toward school, followed by truancy and then mathematics achievement. The significance of indirect effects were checked with 1000 bootstrapping samples with the imputed data and the bias-corrected two-tailed significance showed that all indirect effects were significant except for these of perceived cultural distance to the three outcome variables in Denmark and Slovenia (see Appendix 2, Supplementary Data Sheet, for confidence intervals).

## Discussion

We were interested in the relationship between acculturation orientations, school belonging, and school adjustment (school-related attitudes, truancy, and mathematics achievement) of immigrant students, and how this relationship may differ between countries of settlement, controlling for family educational background, generational status, test language used/not used at home and perceived cultural distance. Drawing on data from PISA 2012, we expected that between-country differences in the associations of acculturation orientations with school belonging and adjustment would reflect differences in multicultural policies (H1). We also expected that links between the acculturation orientations on outcomes would be mediated by sense of school belonging (H2).

We found support for our first hypothesis. There were between-country differences in the effects of the acculturation orientations on school belonging. As expected, a stronger mainstream orientation was associated with a stronger sense of school belonging in all countries, but the additional positive effect of ethnic orientation could only be observed in some countries. As we had anticipated, the variation in successful acculturation strategies seems to be mostly between integration (positive effect of both orientations) and assimilation (positive effect only of mainstream orientation). The effects of both acculturation orientations and the covariates on all three school adjustment outcomes were largely mediated by students’ sense of school belonging, thereby supporting our second hypothesis.

The smallest effect of mainstream orientation was observed in countries with the most inclusive institutional conditions (as measured by the MIPEX) and supportive multicultural policies, notably Belgium and Finland. This suggests that in these countries, there is less pressure to assimilate and therefore it is not as essential to endorse the mainstream culture in order to experience belonging at school. As these countries actively promote a society of cultural pluralism, this may help students who endorse their ethnic culture to also experience belonging at school.

In the “intermediate” countries, Italy and Portugal, there are still rather inclusive institutional conditions, but these are combined with only moderately supportive multicultural policies. This means that these countries capitalize on equity (i.e., promoting access and equal rights of immigrants in school and society), but are not as favorable in terms of actively promoting cultural pluralism. In both countries, students had a higher mainstream than ethnic orientation, suggesting a general tendency of assimilation. We found that a stronger mainstream orientation was also most helpful in order to experience belonging at school in these countries, whereas the orientation toward the ethnic culture was unrelated with school belonging. This pattern suggests that assimilation is the most successful strategy in these “intermediate” countries with their focus on equity. These results also mirror findings of equity-promoting policies in schools supporting assimilation of immigrant students (Schachner et al., 2016).

In the two countries with the least supportive policies, Denmark and Slovenia, the positive effect of mainstream orientation fell in between what was found for the highly supportive and the moderately supportive clusters. This may mean that mainstream orientation is still important in order to experience belonging in school, but at the same time, entry into the mainstream may be more difficult as the societal climate is rather exclusive. A mainstream orientation amongst immigrants may therefore be faced with antagonism by members of the mainstream society, which makes it more difficult to experience belonging in school. In both countries, ethnic orientation is also higher than mainstream orientation, indicating a trend toward separation. Surprisingly, a stronger ethnic orientation was also associated with stronger school belonging in the unsupportive cluster. There are several possible explanations for this: First, this unexpected finding may reflect the presence of a “reactive ethnicity” (Portes and Rumbaut, 2001), an immigrant group’s focus on intra-ethnic identities and relationships that is the result of a hostile climate in the mainstream society or the experience of ethnic segregation. In such non-inclusive environments immigrant students can only get support from their own ethnic group and immigrants focusing more on the own ethnic group can be expected to show more positive associations with healthy functioning, including sense of belonging and general well-being and adjustment at school. A strong ethnic identity may help students deal with discrimination and a generally less welcoming climate in these countries. The second possible explanation is somewhat linked to and supports the first one: As the items measuring sense of belonging at school were mainly tapping into school as a broader social context, feelings of loneliness and happiness as reflected in the items may not necessarily be linked to school as an institution, but could be linked to, amongst others, friends in school, including ethnic friends.

The other surprising finding was that perceived distance had the greatest negative effect on school belonging in countries with the most supportive policies. This may imply that in countries with a supportive policy climate, individual differences in perceived cultural distance make it more difficult for some people to belong than for others. In countries with an unsupportive climate on the other hand, the overall policy climate may be hostile for all immigrant groups, making it difficult to experience belonging for all immigrants, regardless of their level of distance from the mainstream culture.

### Limitations

Even though we were able to draw on a large dataset comprising more than 5,000 students from six OECD countries, our study is not without limitations: First, our analyses are cross-sectional and do not allow any causal interpretations. Second, immigrant groups have their preferred countries of settlement. As a consequence, there is no complete crossing of immigrant groups and countries, which makes it impossible to address the interaction of immigrant group and country of settlement effects. As the country of origin was captured in very different ways across countries, we also could not include this information in our analyses. Our covariates were therefore the only way of controlling for differences in the immigrant populations in the six countries. Third, particularly the unsupportive policy cluster comprised two countries which are very different from each other, also in terms of their immigrant populations (Denmark and Slovenia). As some of the surprising findings emerged from that cluster these should be interpreted with caution. Fourth, the measure of school belonging in the current dataset does not allow for a more fine-grained distinction between school as an institution and school as a social context. More targeted assessments of sources of school belongingness of immigrant students would probably reveal a more detailed and seemingly less counterintuitive picture than found in the present study. Taken together, there are some limitations that arise from measures and data available in the 2012 PISA dataset. These limitations only allowed for *post hoc* explanation for some unexpected findings, which would need to be verified in future research. Nevertheless, the data we could draw on are large and representative within the countries that were sampled and despite these limitations there were several important insights to be gained.

## Conclusion

We highlighted the crucial role of sense of school belonging in linking acculturation orientations and psychological, behavioral and achievement-related school adjustment outcomes across countries. We could also provide interesting insights as to how these associations may vary between countries differing in institutional support for access and equal rights of migrants and multicultural policies. Given the current wave of mass immigration in Europe and the great importance of these policies, our study is of high relevance to policy makers as well as educational practitioners and researchers.

Much educational research and especially large-scale educational surveys such as PISA focussed on achievement and how this is affected by immigrant students’ mainstream language skills. Yet, our study highlights the importance of other, more psychological and behavioral outcomes such as school belonging, attitudes toward school, and truancy, which are related to achievement. Our results suggest that multicultural policies in particular, which are only endorsed by few of the countries in our study, may allow immigrant students to draw on their ethnic culture as well as the mainstream culture as an additional resource for school belonging and adjustment. This finding supports what has been concluded by Ward (2013) in a recent review of acculturation research, namely that integration can only flourish when the context allows. Especially in countries where integration is less promoted on an institutional and policy level, schools may play a crucial role in promoting multicultural values and thereby integration and adjustment of immigrant students (Schachner et al., 2016). Future research should therefore investigate school’s approaches to cultural diversity, as well as other school-level variables such as ethnic composition, and their interplay with institutional conditions and policies at the national level. At the same time, while changes at the policy level may take longer to be implemented and show effects, interventions at the school level may be easier to implement and quicker in showing (positive) effects.

## Author Contributions

MS conceived of the study, was leading its design and coordination, and drafted most of the introduction and the complete discussion of the manuscript. JH participated in the design, performed the statistical analyses, drafted methods and results section and revised the manuscript. BH participated in the design, wrote the section about multicultural policies, was actively involved in the interpretation of the data and revised the manuscript. FV participated in the design, statistical analyses and interpretation of the data and revised the manuscript. All authors read and approved the final manuscript and are accountable for all aspects of the work in ensuring that questions related to the accuracy or integrity of any part of the work are appropriately investigated and resolved.

## Conflict of Interest Statement

The authors declare that the research was conducted in the absence of any commercial or financial relationships that could be construed as a potential conflict of interest.
