# Supplementary material for: Acculturation and School Adjustment of Immigrant Youth in Six European Countries: Findings from the Programme for International Student Assessment (PISA)
Source: Front Psychol. 2017 May 4;8:649. doi: 10.3389/fpsyg.2017.00649 (PMC5415604; doi:10.3389/fpsyg.2017.00649)
Supplement: Supplementary file 1 [file Data_Sheet_1.pdf]

Supplementary Material

Acculturation and School Adjustment of Immigrant Youth in Six European Countries: Findings from the Programme for International Student Assessment (PISA)

Maja K. Schachner* [1], Jia He [2], Boris Heizmann [3], Fons J. R. Van de Vijver [4, 5, 6]

[1] Inclusive Education, University of Potsdam, Potsdam, Germany

[2] Department of Educational Quality and Evaluation, German Institute for International Educational Research, Frankfurt am Main, Germany

[3] Department Data Archive for the Social Sciences, GESIS – Leibniz Institute for the Social Sciences, Cologne, Germany

[4] Department of Culture Studies, Tilburg University, Tilburg, the Netherlands

[5] Faculty of Economics and Management Sciences, North-West University, Potchefstroom, South Africa

[6] School of Psychology, University of Queensland, Brisbane, Australia

*** Correspondence:**

Maja Katharina Schachner
E-Mail: maja.schachner@uni-potsdam.de
Secondary E-Mail: maja.schachner@googlemail.com

Appendix 1. Correlations between study variables across the whole sample.

	Male	1st-Generation Immigrant	Test Language Use at Home	Family Educational Background	Host orientation	Heritage orientation	Perceived Cultural Distance	School Belonging	Math Achievement (PV3)	Truancy	Attitude towards School
Male	1										
1st-Generation Immigrant	-.04**	1									
Test Language Use at Home	.06**	-.19**	1								
Family Educational Background	.06**	-.02	.06**	1							
Host orientation	.02	.10**	.09**	.12**	1						
Heritage orientation	.05**	-.02	-.16**	-.05**	.14**	1					
Perceived Cultural Distance	-.06**	.09**	.05**	.01	.31**	.08**	1				
School Belonging	.05**	-.16**	.11**	-.01	.22**	.22**	.14**	1			
Math Achievement (PV3)	-.05**	-.05**	.03*	.27**	.14**	-.06**	-.05**	.11**	1		
Truancy	-.03*	.05**	.00	-.05**	-.04**	-.02	-.02	-.18**	-.21**	1	
Attitude towards School	.11**	-.02	.01	.02	.20**	.14**	.15**	.48**	.16**	-.23**	1

Note. ** p <.01. * p < .05.

Appendix 2. 95% Confidence Intervals of standardized indirect effects.

	First-generation Immigrants		Percieved Cultural Distance		Test Language at Home		Heritage Orientation		Host Orientaion	
Belgium	lower	upper	lower	upper	lower	upper	lower	upper	lower	upper
Attitude to School	-.067	-.040	.055	.098	.028	.055	.078	.120	.039	.085
Math Achievement pv3	-.019	-.010	.014	.027	.007	.014	.018	.032	.009	.023
Truancy	.017	.031	-.045	-.024	-.025	-.012	-.054	-.033	-.038	-.017
Denmark										
Attitude to School	-.063	.003	-.017	.635	.028	.002	.085	.002	.079	.002
Math Achievement pv3	-.020	.002	-.005	.632	.007	.002	.022	.001	.021	.001
Truancy	.014	.002	-.010	.628	-.022	.001	-.052	.002	-.051	.002
Finland										
Attitude to School	-.072	-.044	.056	.099	.026	.050	.085	.132	.036	.081
Math Achievement pv3	-.020	-.010	.013	.027	.006	.013	.020	.035	.009	.022
Truancy	.016	.028	-.038	-.020	-.019	-.009	-.050	-.030	-.030	-.013
Italy										
Attitude to School	-.059	-.036	.018	.065	.028	.054	.029	.074	.100	.149
Math Achievement pv3	-.016	-.008	.004	.016	.006	.013	.007	.018	.022	.038
Truancy	.012	.022	-.024	-.006	-.020	-.010	-.027	-.010	-.056	-.034
Portugal										
Attitude to School	-.073	-.044	.017	.065	.032	.062	.031	.082	.115	.172
Math Achievement pv3	-.019	-.009	.004	.016	.007	.015	.007	.020	.024	.044
Truancy	.014	.025	-.022	-.006	-.021	-.010	-.028	-.010	-.059	-.035
Slovenia										
Attitude to School	-.067	-.040	-.017	.027	.030	.060	.085	.133	.068	.110
Math Achievement pv3	-.019	-.010	-.004	.007	.008	.016	.021	.039	.017	.033
Truancy	.012	.022	-.008	.005	-.019	-.009	-.042	-.025	-.035	-.020
